# Mapping MAGE-A4 expression in solid cancers for targeted therapies

**DOI:** 10.3389/fonc.2025.1484182

**Published:** 2025-03-13

**Authors:** Christin Habigt, Sylvie Rottey, Iben Spanggaard, Juanita S. Lopez, Elena Garralda, Emiliano Calvo, Oliver Bechter, Jayesh Desai, Rachel Galot, Leena Gandhi, Florian Heil, Natascha Rieder, Ivan Dimitrov, Iris Martinez Quetglas, Christian Heichinger, Nino Keshelava, Andreas Roller

**Affiliations:** ^1^ Roche Pharma Research and Early Development, Early Development Oncology, Roche Innovation Center Munich, Penzberg, Germany; ^2^ Department of Medical Oncology, UZ Gent, Ghent, Belgium; ^3^ Department of Oncology, Copenhagen University Hospital, Rigshospitalet, Copenhagen, Denmark; ^4^ Phase I Drug Development Unit, The Institute of Cancer Research and The Royal Marsden Hospital NHS Foundation Trust, London, United Kingdom; ^5^ Institute of Oncology (VHIO), Vall d’Hebron University Hospital, Barcelona, Spain; ^6^ START Madrid-CIOCC, Centro Integral Oncológico Clara Campal, Madrid, Spain; ^7^ Department of General Medical Oncology, University Hospitals Leuven, Leuven, Belgium; ^8^ Department of Oncology, KU Leuven, Leuven, Belgium; ^9^ Peter MacCallum Cancer Centre, Melbourne, VIC, Australia; ^10^ Université Catholique de Louvain and Institut Roi Albert II, Cliniques Universitaires Saint-Luc, Brussels, Belgium; ^11^ Early Drug Development Center, Dana-Farber Cancer Institute, Boston, MA, United States; ^12^ CDx Pharma Services Assay Development, Roche Tissue Diagnostics, Tucson, AZ, United States; ^13^ Roche Pharma Research and Early Development, Early Development Oncology, Roche Innovation Center Zurich, Zurich, Switzerland; ^14^ Roche Pharma Research and Early Development, Early Development Oncology, Roche Innovation Center Basel, Basel, Switzerland

**Keywords:** biomarker, translational analysis, clinical trial, target expression, patient selection

## Abstract

Melanoma-associated antigen A4 (MAGE-A4) is a promising target for anticancer therapy. However, limited contemporary data are available on the details of MAGE-A4 protein expression in different cancer types. In this study, the protein expression of MAGE-A4 is comprehensively studied in patients with unresectable and/or metastatic solid cancers to identify indications of the highest unmet medical need for anti-MAGE-A4 therapy. FFPE tumor sections from 200 patients, predominantly HLA-A*02:01 positive (*n* = 193), were examined using immunohistochemistry (IHC) to detect MAGE-A4 expression. The patient cohort comprised various cancer types to pinpoint differences in the prevalence and intensity of MAGE-A4 positivity. MAGE-A4 expression was observed in 35% (69 patients) of the overall cohort. Certain cancer types exhibited notably higher frequencies of MAGE-A4 positivity. Specifically, adenoid cystic carcinoma demonstrated the highest prevalence at 82%, followed by liposarcoma at 67%. Ovarian serous/high-grade carcinoma showed a 64% positivity rate, identical to that observed in squamous non-small cell lung cancer (NSCLC). Head and neck squamous cell carcinoma (HNSCC) presented a 60% prevalence, while esophageal cancer had a 54% prevalence of MAGE-A4 expression. These data highlight the variability of MAGE-A4 expression across different cancer types and underscore its relevance as a potential target of novel precision medicines. The significant presence of MAGE-A4 in specific cancers suggests potential for stratified therapeutic approaches and warrants further investigation into its role in oncogenesis and treatment response.

## Introduction

Cancer testis antigens (CTAs) are proteins with a restricted expression pattern that is limited to germ cells and trophoblasts in healthy adults and tumor cells in patients with cancer (1, 2). When expressed in cancer cells, CTAs are considered foreign antigens and have the capacity to elicit adaptive immune responses that are cancer-specific. For these reasons, CTAs are considered attractive targets for cancer immunotherapy (1–3).

Melanoma-associated antigen A4 (MAGE-A4) is a member of the melanoma-associated antigen family of CTAs (4). Intracellular expression of MAGE-A4 has been observed in several tumor types (5, 6), with ≥20% prevalence reported in synovial sarcoma and myxoid/round cell liposarcoma, bladder urothelial carcinoma, gastric cancer, ovarian carcinoma, esophageal cancer, and head and neck squamous cell carcinoma (HNSCC) (6). In cancer cells, MAGE-A4 proteins are processed into peptide fragments that are subsequently presented by human leukocyte antigens (HLAs) at the cell surface in a form that can be recognized by circulating T cells (7), leading to T-cell activation and cytolytic activity (4). Beyond its role as a tumor antigen, MAGE-A4 has been implicated in cancer progression, aggressiveness, and metastasis. Studies suggest that MAGE-A4 can promote tumor cell survival by inhibiting apoptosis and enhancing proliferation. Additionally, MAGE-A4 may contribute to increased metastatic potential by influencing epithelial–mesenchymal transition (EMT) and enhancing cell migration, further supporting its role as a potential therapeutic target in aggressive cancers (8, 9). Given these characteristics, MAGE-A4 has been targeted in several recent clinical studies evaluating novel immunotherapies against solid cancers, with manageable safety and promising activity reported (7, 10, 11). Notably, the recent FDA approval of a MAGE-A4-targeted therapy demonstrates its potential as a viable therapeutic target in oncology (12). Despite this progress, contemporary data on the prevalence of MAGE-A4 protein expression across solid tumors remain limited, presenting an important area for further research to support the advancement of emerging MAGE-A4-targeted therapies.

## Methods

### Patient cohort

MAGE-A4 protein expression was evaluated in tumor sections obtained from a total of 213 patients, with samples from 200 of these patients being evaluable for MAGE-A4 protein assessment. Out of the 200 patients, 102 were male and 98 were female patients. Archival or fresh FFPET tissue sections were obtained from patients with unresectable and/or metastatic solid cancers who were screened for enrollment into an open-label, multicenter, phase I study (NCT05129280), targeting the MAGE-A4 230–239 peptide (GVYDGREHTV) in the context of HLA-A*02:01. The inclusion criteria were as follows: age ≥18 years, unresectable and/or metastatic solid tumors, received prior standard of care (SOC) treatment and no subsequent SOC treatment available, and measurable disease according to the Response Evaluation Criteria in Solid Tumors v1.1. The protocol for this study was approved by the responsible institutional review boards/ethics committees for each center (see **Supplementary Table 1**); informed consent was obtained before any screening evaluations were carried out.

### HLA typing

For patients where the information had not yet been obtained previously at the site by a validated methodology, HLA-A*02:01 allele typing was performed centrally with a validated methodology at a CAP/CLIA-certified laboratory (Laboratory Corporation of America Holdings, Burlington, NC, USA). First, DNA was extracted with a commercial kit (QIAGEN, Germantown, MD, USA) from either buccal swab or blood samples. Then, the extracted DNA was analyzed using 1) a validated laboratory-developed test (LDT) for Sanger sequencing of exons 2 and 3 and (2) a commercial kit (CareDx, Brisbane, CA, USA) for sequencing of exons 1 through 4 to achieve a higher resolution typing for all specimens that carry A*02:01 genotypes. Details on the LDT can be found in the **Supplementary Material**. Commercially available HLA typing software (Assign software) was used to convert sequence files to the corresponding HLA genotypes.

### MAGE-A4 IHC

MAGE-A4 protein expression was centrally assessed in a CAP/CLIA-certified laboratory (Roche Tissue Diagnostics, Tucson, AZ, USA) in sectioned archival or fresh formalin-fixed paraffin-embedded tumor (FFPET) tissue, using an investigational MAGE-A4 immunohistochemistry (IHC) assay with mouse monoclonal clone OTI1F9 as the primary antibody (OriGene Technologies, Inc., Rockville, MD, USA) and employing the BenchMark ULTRA system and OptiView DAB IHC detection kit (Roche Tissue Diagnostics, Tucson, AZ, USA). The percentage of tumor cells with no MAGE-A4 expression or a MAGE-A4 staining intensity of level 1+ (weak), 2+ (moderate), or 3+ (strong) was visually assessed by board-certified pathologists as one combined score for cytoplasmic and nuclear staining. Tumor sections with at least 1% of tumor cells with a level of at least 1+ MAGE-A4 staining intensity were considered MAGE-A4 positive. For more detailed information on the MAGE-A4 IHC assay, please refer to the **Supplementary Material**.

The histo-score (*H*-score) was calculated using the formula: 3× the percentage of tumor cells with level 3+ staining intensity + 2× the percentage of tumor cells with level 2+ intensity + 1× the percentage of tumor cells with level 1+ intensity, giving a range of 0 to 300.

### B2M and MHC IHC

Beta-2-microglobulin (B2M) and major histocompatibility complex (MHC) class I expression were also centrally assessed using exploratory IHC assays in tumor sections obtained from a subset of patients who were MAGE-A4 and HLA-A*02:01 positive and enrolled into the phase I trial. For both assays, membranous and cytoplasmic staining was visually assessed by board-certified pathologists as one combined score. Tumor sections with ≥50% tumor cells with a level of at least 1+ B2M or MHC class I staining intensity were considered B2M or MHC class I positive, respectively, as previously described (13).

## Results

Archival or fresh FFPET tissue sections were received from a total of 213 patients during screening; 200 tumor samples were evaluable for MAGE-A4 protein expression. Overall, tumor samples from 193 patients were HLA-A*02:01 positive, two were HLA-A*02:01 negative, and five had HLA-A*02:01 of unknown status (not tested). The median age of these patients was 59 years (range: 26–84) and most (96%) were Caucasian. Common indications were colorectal cancer (*n* = 22); pancreatic cancer (*n* = 16); breast cancer (*n* = 15); ovarian serous/high-grade carcinoma (*n* = 14); sarcoma, diverse (*n* = 14); esophageal cancer (*n* = 13); adenoid cystic carcinoma (*n* = 11); and squamous NSCLC (*n* = 11; **Table 1**). In a preliminary analysis of pre-existing tumor bulk RNA-seq data sets, HLA-A*02:01 status had been found to not correlate with tumor MAGE-A4 expression (data not shown). The median time from tumor biopsy to MAGE-A4 protein expression assessment was 12 months (range: 1 week–13 years). Adequate MAGE-A4 staining quality was observed in FFPET tissue sections, regardless of sample age (**Supplementary Figure 1**).

**Table 1:** Prevalence of MAGE-A4 expression by indication.

Indication	Patients tested, *N*	Patients with MAGE-A4-positive tumor cells, *n* (%)	Average MAGE-A4-positive tumor cells^a^, % (range)	Average *H*-score^a^ (range)
Adenoid cystic carcinoma	11	9 (82)	49 (2–98)	118 (4–282)
Liposarcoma	6	4 (67)	59 (10–80)	99 (11–155)
Ovarian serous/high-grade carcinoma	14	9 (64)	34 (1–98)	61 (1–203)
Squamous NSCLC	11	7 (64)	79 (25–100)	169 (50–275)
HNSCC	5	3 (60)	22 (1–40)	31 (2–65)
Esophageal cancer	13	7 (54)	64 (3–100)	155 (5–300)
Vulvar carcinoma	2	1 (50)	23 (NA)	41 (NA)
Ovarian carcinoma, other	9	4 (44)	61 (2–100)	141 (2–295)
Gastric cancer	7	3 (43)	78 (65–100)	131 (70–215)
Endometrial cancer	5	2 (40)	29 (7–50)	51 (12–90)
Synovial sarcoma	6	2 (33)	99 (98–100)	213 (196–230)
Non-NSCLC lung cancer	3	1 (33)	1 (NA)	2 (NA)
Pancreatic cancer	16	5 (31)	30 (2–85)	48 (4–160)
Bladder urothelial carcinoma	9	2 (22)	6 (2–8)	10 (4–16)
Melanoma	5	1 (20)	10 (NA)	20 (NA)
Colorectal cancer	22	3 (14)	13 (5–25)	21 (10–35)
Breast cancer	15	2 (13)	36 (16–55)	82 (23–140)
Sarcoma, diverse	14	1 (7)	2 (NA)	3 (NA)
Renal cell carcinoma	5	0	NA	NA
Mesothelioma	3	0	NA	NA
Cholangiocarcinoma	2	0	NA	NA
NSCLC adenocarcinoma/undefined	2	0	NA	NA
Prostate cancer	2	0	NA	NA
Chordoma	2	0	NA	NA
Other^b^	11	3 (27)	78 (60–95)	155 (80–281)
**All indications**	**200**	**69 (35)**	**48 (1–100)**	**99 (1–300)**

HNSCC, head and neck squamous cell carcinoma; MAGE-A4, melanoma-associated antigen A4; NA, not applicable; NSCLC, non-small cell lung cancer.

a In patients who are MAGE-A4 positive.

b Details for “other” indications can be found in **Supplementary Table 3**.

Of the 200 evaluable patients, tumor samples from 69 (35%) patients were MAGE-A4 positive (prevalence data by indication are presented in **Table 1**). Overall, the prevalence and abundance of MAGE-A4 positivity was comparable among tumor samples collected from primary (33%) and metastatic (35%) sites (**Supplementary Table 4** provides detailed information on all samples analyzed in this study). Among the positive cases, the percentage of MAGE-A4-positive cells was similar between primary and metastatic sites (**Supplementary Figure 2A**). However, there were differences between metastatic sites; lung (62%), lymph node (50%), peritoneum/abdominal wall (50%), and soft tissue (50%) metastases were commonly MAGE-A4 positive, whereas liver metastases were mostly MAGE-A4 negative (17% positivity) (**Supplementary Table 2**). One patient with colorectal carcinoma provided two evaluable tumor samples, one from the primary tumor and one from a metastasis; both samples were MAGE-A4 negative (**Supplementary Table 4**). The prevalence and abundance of MAGE-A4 positivity was also comparable for men (33%) and women (36%; **Supplementary Table 4** provides detailed information on all samples analyzed in this study). Among the positive cases, the percentage of MAGE-A4-positive cells was similar between male and female patients (**Supplementary Figure 2B**). Notably, indications with ≥5 evaluable patients and ≥50% prevalence of MAGE-A4 positivity were adenoid cystic carcinoma (*n* = 9/11; 82%), liposarcoma (*n* = 4/6; 67%), ovarian serous/high-grade carcinoma (*n* = 9/14; 64%), squamous NSCLC (*n* = 7/11; 64%), HNSCC (*n* = 3/5; 60%), and esophageal cancer (*n* = 7/13; 54%). Indications with ≥5 evaluable patients and <50% MAGE-A4 positivity were ovarian carcinoma; other (excluding serous/high-grade carcinoma; *n* = 4/9; 44%); gastric cancer (*n* = 3/7; 43%); endometrial cancer (*n* = 2/5; 40%); synovial sarcoma (*n* = 2/6; 33%); pancreatic cancer (*n* = 5/16; 31%); bladder urothelial carcinoma (*n* = 2/9; 22%); melanoma (*n* = 1/5; 20%); colorectal cancer (*n* = 3/22; 14%); breast cancer (*n* = 2/15; 13%); sarcoma, diverse (*n* = 1/14; 7%); and renal cell carcinoma (*n* = 0/5; **Table 1**). In the subset of MAGE-A4- and HLA-A*02:01-positive patients that enrolled in the phase I trial (*n* = 19), 12/19 (63%) patients were positive for both MHC class I and B2M, 5/19 (26%) patients were positive for MHC class I but negative for B2M, and 2/19 (11%) were negative for both markers (**Supplementary Figure 3**). Neither MHC class I nor B2M expression correlated with MAGE-A4 expression (data not shown).

Among all MAGE-A4-positive tissue sections, the average percentage of MAGE-A4-positive tumor cells was 48% (range: 1–100; **Table 1**). Indications with ≥3 MAGE-A4-positive patients and an average percentage of MAGE-A4-positive tumor cells of ≥30% were squamous NSCLC (79%), gastric cancer (78%), esophageal cancer (64%), ovarian carcinoma, other (61%), liposarcoma (59%), adenoid cystic carcinoma (49%), ovarian serous/high-grade carcinoma (34%), and pancreatic cancer (30%; **Table 1**).

**Figure 1:**
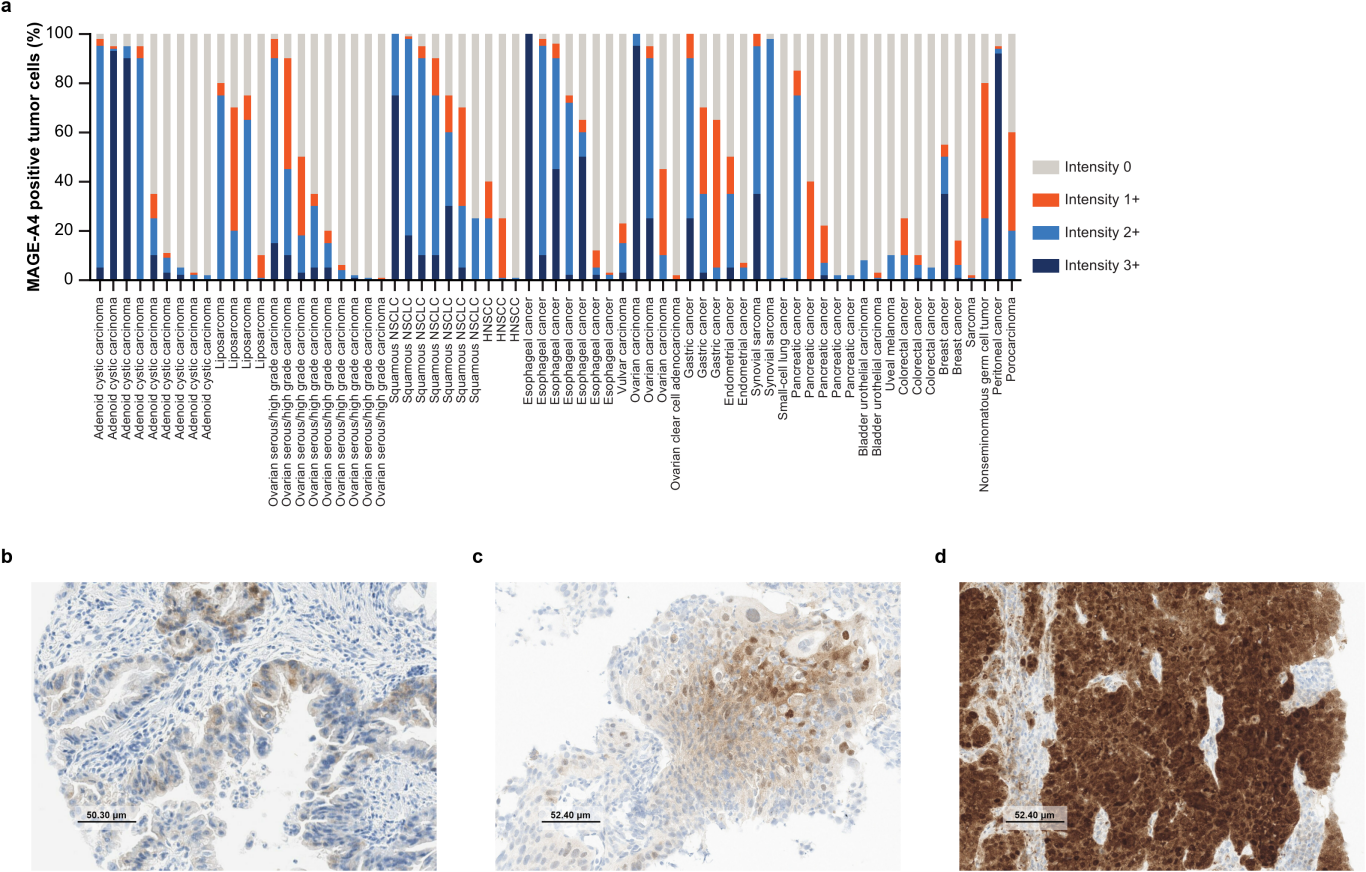
Abundance of MAGE-A4 expression. **(a)** Distribution of cyto/nuclear MAGE-A4 staining intensity by indication. Each bar represents one patient. **(b)** Example IHC image of a MAGE-A4-positive primary gastric adenocarcinoma biopsy with a cytoplasmic staining pattern, 65% positive tumor cells (60% at 1+ intensity), and *H*-score of 70. **(c)** Example IHC image of a MAGE-A4-positive primary squamous NSCLC biopsy with a mixed staining pattern, 70% positive tumor cells (40% at 1+ intensity; 25% at 2+ intensity), and *H*-score of 104. **(d)** Example IHC image of a MAGE-A4-positive lymph node metastatic biopsy of peritoneal cancer with a nuclear staining pattern, 95% positive tumor cells (92% at 3+ intensity), and *H*-score of 281. **(b–d)** Taken at ×20 original magnification; MAGE-A4 staining intensity was scored level 1+ (weak), 2+ (moderate), or 3+ (strong). HNSCC, head and neck squamous cell carcinoma; IHC, immunohistochemistry; *H*-score, histo-score; MAGE-A4, melanoma-associated antigen A4; NSCLC, non-small cell lung cancer.

Information on the distribution of MAGE-A4 staining intensity by indication is presented in **Figure 1a**. Among all MAGE-A4-positive tissue sections, the average *H*-score was 99 (range: 1–300). Average *H*-scores by indication are presented in **Table 1**, with other indications detailed in **Supplementary Table 3** and full sample information on all tested patients detailed in **Supplementary Table 4**. Indications with ≥3 MAGE-A4-positive patients and a high-intensity average *H*-score (201–300) were synovial sarcoma (average score 213); those with a moderate-intensity average *H*-score (101–200) were squamous NSCLC (169), esophageal cancer (155), ovarian carcinoma, other (141), gastric cancer (131), and adenoid cystic carcinoma (118); and those with a low-intensity average *H*-score (1–100) were liposarcoma (99), ovarian serous/high-grade carcinoma (61), pancreatic cancer (48), HNSCC (31), and colorectal cancer (21). **Figures 1b–d** show representative images of sections of tumors stained by IHC for MAGE-A4.

## Discussion

In the current study, protein expression of MAGE-A4 in FFPET tissue sections was evaluated that originated from 200 patients who had unresectable and/or metastatic solid cancers and who were mostly (*n* = 193) HLA-A*02:01 positive. The overall prevalence of MAGE-A4 protein expression across indications was 35% (**Table 1**). MAGE-A4 positivity appeared to be particularly prevalent (>50% prevalence) and abundant (average percentage of MAGE-A4 tumor cells ≥ 30%) at the same time in tumor sections from patients with adenoid cystic carcinoma, liposarcoma, ovarian serous/high-grade carcinoma, squamous NSCLC, and esophageal cancer, with a moderate-intensity average *H*-score in the squamous NSCLC, esophageal cancer, and adenoid cystic carcinoma indications. Prevalence of at least 35% at an average abundance of at least 30% MAGE-A4-positive tumor cells was also found for ovarian carcinoma, other (excluding serous/high-grade carcinoma; 44% prevalence, average of 61% positive tumor cells), and gastric cancer (43% prevalence, average of 78% positive tumor cells). Notably, while the prevalence of MAGE-A4 was determined to be below average for synovial sarcoma (33%) in this study, in all cases when it was detected, abundance was uniformly very high with 98% and more tumor cells being positive for MAGE-A4 and high *H*-scores of 196 and above (**Table 1**).

In a subgroup of 19 HLA-A*02:01- and MAGE-A4 double-positive patients, most tumors (63%) were also positive for MHC class I and B2M (**Supplementary Figure 3**), indicating that these tumors are competent in antigen presentation which is crucial when targeting intracellular proteins through cancer immunotherapy. Collectively, these data suggesting high MAGE-A4 prevalence and/or abundance in several tumor types may help to inform the direction of future research programs that are targeting the MAGE-A4 antigen in patients with unresectable and/or metastatic solid cancers.

The prevalence of MAGE-A4 expression in solid tumors has been evaluated in two recent studies. However, cross-study comparisons are complicated by inconsistent methodology. In the first study, Wang and coworkers (6) evaluated the prevalence of MAGE-A4 protein expression in 1,750 tumor sections obtained from patients with any HLA-A*02 allele (except HLA-A*02:05P) using an investigational IHC assay with the same antibody clone (OTI1F9) as in this study. MAGE-A4 positivity was defined by a cutoff of ≥30% tumor cell staining at ≥2+ staining intensity. The overall prevalence of MAGE-A4 positivity was 20%. Indications with ≥20% prevalence of MAGE-A4 positivity were synovial sarcoma (70%), myxoid/round cell liposarcoma (40%), urothelial cancer (32%), esophagogastric junction cancer (26%), ovarian cancer (24%), HNSCC (22%), and esophageal cancer (21%). When using the same cutoff to analyze the data from the current study, overall prevalence of MAGE-A4 positivity was similar (15.5%), while indications with ≥20% prevalence of MAGE-A4 positivity were squamous cell NSCLC (55%), gastric cancer (43%), esophageal cancer (38%), adenoid cystic carcinoma (36%), liposarcoma (33%), synovial sarcoma (33%), ovarian cancer (21% for serous/high grade, 22% for other), and endometrial cancer (20%; refer to **Supplementary Table 4** for information on all samples analyzed in this study). In the second study, Ishihara and colleagues (5) evaluated the prevalence of MAGE-A4 gene expression in 585 tissue samples using a real-time polymerase chain reaction assay. The overall prevalence of MAGE-A4 gene expression was the same as observed in our study (35%); MAGE-A4 positivity was particularly prevalent (≥20% of samples) in esophageal cancers (55%), head and neck cancers (38%), gastric cancers (35%), and ovarian cancers (34%).

As in the present study, MAGE-A4 expression was found to be similar between primary tumor and metastatic samples (**Supplementary Table 4**, **Supplementary Figure 2A**). Upon investigating MAGE-A4/A9 expression in high-risk bladder cancer, Bergeron and colleagues (14) compared 493 primary bladder tumors and 33 lymph node metastatic samples, of which only four samples were discordant (three samples were MAGE-A4/A9 positive in the lymph node but MAGE-A4/9 negative in the primary tumor, and one sample was vice versa). Additionally, MAGE-A3/A4 expression was positive in 39% of primary tumors, 42% of lymph node metastases, and 37% of tumor recurrences when observing MAGE-A3/A4 expression in patients with head and neck squamous cell carcinoma (15). Strikingly, in a study of primary and recurrent vulvar cancer, Bellati et al. (16) found that tumors with lymph node metastases had higher MAGE-A4 expression than those without, providing a possible explanation for the higher rate of MAGE-A4 positivity found in metastatic lymph node samples versus primary tumors observed in our study (**Supplementary Tables 2**, **4**).

IHC as a method for assessing tumor biomarker expression has notable limitations, especially when evaluating targets like MAGE-A4. One key limitation is that marker assessment with IHC relies on a small sampling area of a single lesion for each patient (such as a single core needle biopsy), which may not fully capture the complexity of tumor heterogeneity, particularly in very advanced disease settings. Tumors can also exhibit substantial variability both within a single lesion (intratumor heterogeneity) and across different tumor sites (intertumor heterogeneity), which may result in an incomplete or inconsistent representation of biomarker expression. Additionally, there is an inherent dilemma in measuring MAGE-A4 presence with IHC, as this method detects only the MAGE-A4 protein rather than the actual peptide/MHC complex that serves as the therapeutic target. This indirect measurement means that positive IHC results for MAGE-A4 may not accurately represent the presence or functional stability of the peptide/MHC complex in tumor cells, introducing further complexity into the interpretation of results. Finally, visual IHC scoring involves a degree of subjectivity, introducing reader-dependent variability that can impact the interpretation and reliability of results. In particular, the scoring of staining intensities, which are the basis of the calculation of the *H*-score, is prone to subjectivity.

Our findings provide important insights into MAGE-A4 expression in solid cancers. However, the extent to which these findings can be generalized to the broader population of patients with diverse tumors remains to be fully established. While our cohort reflects MAGE-A4 expression in solid cancers, variability in patient demographics and genetic profiles should be considered. Further studies in larger and more diverse populations are needed to confirm the broader applicability of these observations. Nonetheless, these findings contribute to the growing body of evidence indicating a high prevalence of MAGE-A4 expression in several tumor types with unmet medical needs. These data may help guide future research efforts evaluating MAGE-A4-targeted therapies in patients with solid tumors.

## Data Availability

The raw data supporting the conclusions of this article will be made available by the authors without undue reservation.
