# Supplementary material for: Mapping MAGE-A4 expression in solid cancers for targeted therapies
Source: Front Oncol. 2025 Mar 13;15:1484182. doi: 10.3389/fonc.2025.1484182 (PMC11947667; doi:10.3389/fonc.2025.1484182)
Supplement: Supplementary file 1 [file DataSheet1.docx]

**Supplementary materials**

**Supplementary methods:**

**Human leukocyte antigen (HLA) typing: LDT details**

The first step of HLA typing involved a validated LDT for Sanger Sequencing of exons 2 and 3 which code for the HLA Class I Antigen Recognition Domain [ARD]. For this LDT, standard polymerase chain reaction was performed using a validated proprietary primer set to provide amplicons with full coverage of exons 2 and 3. The amplicons were then subjected to Sanger sequencing reactions using the applied Biosystems “Big Dye” terminator chemistry and Capillary Electrophoresis Analyzers (ABI 3130XL and ABI 3730XL). Proprietary sequencing primers were used to provide redundant forward and reverse coverage of both exons 2 and 3 including a panel of group specific sequencing primers designated to resolve common linkage phase ambiguities.

**MAGE-A4 IHC: Background information**The MAGE-A4 IHC assay was optimized and validated across five cancer indications: squamous non-small cell lung cancer; esophageal cancer; ovarian carcinoma; head-and-neck squamous cell carcinoma; synovial sarcoma; and bladder urothelial carcinoma. The optimization process included testing for and determining optimal parameters for antibody titration, antibody diluent selection, epitope retrieval, and primary antibody incubation to achieve a robust, high-intensity signal with minimal background interference. Each condition was refined in consultation with the lead pathologist to ensure diagnostic accuracy and reproducibility. After optimization, the assay was evaluated for robustness and validated before transitioning into a CAP/CLIA-certified laboratory for clinical sample testing.

Prior to sample evaluation, the lead pathologist prepared a standardized training presentation and conducted a comprehensive hour-long introduction to the MAGE-A4 marker for all pathologists involved in scoring. This training session ensured pathologists were aligned on a consistent scoring methodology and set clear guidelines for identifying staining intensity variations. The criteria for scoring emphasized the classification of staining intensity as weak, moderate or strong, which was reinforced through a combined cyto-nuclear scoring approach. This approach emphasizes the most dominant staining compartment within a cell. For example, if one compartment showed a 2+ score and another showed a 3+, the cell was classified as 3+.

For quality control, each staining run included a system-level control (SLC) slide (a validated tissue slide used to confirm uniform staining across the assay). The SLC slide was evaluated before clinical sample staining to ensure that the staining intensity matched previous runs, therefore validating assay stability. Additionally, each sample was stained with an isotype control antibody as a negative control to confirm that staining was specific to the primary antibody and free from non-specific interactions.

Three trained pathologists, each well-versed in the biomarker scoring and cutoff criteria, conducted independent evaluations of the clinical samples. The pathologists achieved a general overall percent agreement (OPA) target of 85% in similar assays, though no formal concordance study was conducted for this assay. Pathologists were permitted to consult with one another to reach consensus on interpretation as needed, although these interactions were not formally recorded in the internal system.

The assay is available for use by any party through the Roche CDx CAP/CLIA laboratory.

**Supplementary Table 1** **⎜Ethics committees.**

| **Study identifier** | **Study Primary Investigator’s ethics approval board/committee** | **City and country** |
| --- | --- | --- |
| NCT05129280 | Peter MacCallum Cancer Centre Ethics Committee | Melbourne, Victoria, Australia |
|  | De Videnskabsetiske Komitéer for Region Hovedstade | Hillerød, Denmark |
|  | CEIC Hospital Vall D'Hebron | Barcelona, Spain |
|  | North West – Greater Manchester (GM) Central | Manchester, United Kingdom |
|  | Western Institutional Review Board-Copernicus Group | Washington, United States of America |
|  | Dana Farber Cancer Inst. Office for Human Research Studies | Boston, United States of America |
|  | Independent Belgian Ethics Committee* | Leuven, Belgium |

*submitted within the Clinical Trial Regulation (CTR) pilot project

**Supplementary Table 2** **⎜Prevalence of MAGE-A4 expression by metastatic lesion site.**

| **Metastatic lesion site** | **Patients tested, *N*** | **Patients with MAGE-A4-positive tumor cells, *n* (%)** | **Average MAGE-A4-positive tumor cells*, % (range)** | **Average *H*-score* (range)** |
| --- | --- | --- | --- | --- |
| **Lung** | 13 | 8 (62) | 66 (3–100) | 140 (5–280) |
| **Lymph node** | 14 | 7 (50) | 72 (23–98) | 151 (41–281) |
| **Peritoneum or abdominal wall** | 8 | 4 (50) | 52 (2–100) | 128 (2–295) |
| **Soft tissue** | 6 | 3 (50) | 37 (2–75) | 71 (3–140) |
| **Liver** | 23 | 4 (17) | 26 (2–85) | 48 (4–160) |
| **Other gastrointestinal location** | 3 | 2 (67) | 51 (1–100) | 151 (1–300) |
| **Kidney** | 3 | 1 (33) | 2 (NA) | 4 (NA) |
| **Ovary** | 3 | 0 | NA | NA |
| **Skin** | 3 | 0 | NA | NA |
| **Other/site not recorded** | 20 | 5 (25) | 35 (2–96) | 68 (4–231) |
| **All metastatic lesion sites** | **96** | **34 (35)** | **51 (1–100)** | **110 (1–300)** |

*In patients who are MAGE-A4 positive with metastatic lesion sites.

MAGE-A4, melanoma-associated antigen A4; NA, not applicable. *H*-score, histo-score.

**Supplementary Table 3 ⎜Prevalence of MAGE-A4 expression by indication – other indications.**

| **Indication** | **Patients tested, N** | **Patients with MAGE-A4-positive tumor cells, *n* (%)** | **MAGE-A4-positive tumor cells*, %** | ***H*-score*** |
| --- | --- | --- | --- | --- |
| **Nonseminomatous germ cell tumor** | 1 | 1 (100) | 80 | 105 |
| **Peritoneal cancer** | 1 | 1 (100) | 95 | 281 |
| **Porocarcinoma** | 1 | 1 (100) | 60 | 80 |
| **Cervical cancer** | 1 | 0 (0) | NA | NA |
| **Colorectal neuroendocrine carcinoma** | 1 | 0 (0) | NA | NA |
| **Duodenal cancer** | 1 | 0 (0) | NA | NA |
| **Esthesioneuroblastoma** | 1 | 0 (0) | NA | NA |
| **Large cell neuroendocrine tumor** | 1 | 0 (0) | NA | NA |
| **Laryngeal neuroendocrine carcinoma** | 1 | 0 (0) | NA | NA |
| **Pancreatic neuroendocrine tumor** | 1 | 0 (0) | NA | NA |
| **Squamous cell carcinoma of the lacrimal drainage system** | 1 | 0 (0) | NA | NA |

*In patients who are MAGE-A4 positive; MAGE-A4, melanoma-associated antigen A4; NA, not applicable. *H*-score, histo-score.

**Supplementary Table 4 ⎜Full data set of MAGE-A4 expression testing.**

| **#** | **Patient age, years** | **Sex** | **Ethnicity** | **Tumor indication** | **Sample type** | **Site of metastasis** | **Sample age, days** | **Tumor cell**  **intensity, %** | | | **Average *H-score*** |
| --- | --- | --- | --- | --- | --- | --- | --- | --- | --- | --- | --- |
|  |  |  |  |  |  |  |  | **1** | **2** | **3** |  |
| **1** | 75 | Female | Caucasian | Ovarian serous/high grade carcinoma | Metastatic | Peritoneum/ abdominal wall | 7 | 8 | 75 | 15 | 203 |
| **2** | 73 | Female | Caucasian | Esophageal cancer | Metastatic | Head and neck | 896 | 6 | 45 | 45 | 231 |
| **3** | 62 | Male | Caucasian | Mesothelioma | Metastatic | Peritoneum/ abdominal wall | 22 | 0 | 0 | 0 | 0 |
| **4** | 62 | Male | Caucasian | Squamous NSCLC | Primary | NA | 785 | 1 | 80 | 18 | 215 |
| **5** | 50 | Male | Caucasian | Squamous NSCLC | Primary | NA | 89 | 0 | 25 | 0 | 50 |
| **6** | 45 | Female | Caucasian | Breast cancer | Metastatic | Liver | 851 | 10 | 5 | 1 | 23 |
| **7** | 55 | Male | Caucasian | HNSCC | Primary | NA | 13 | 24 | 1 | 0 | 26 |
| **8** | 79 | Female | Caucasian | Ovarian serous/high grade carcinoma | Metastatic | Colon | 1928 | 1 | 0 | 0 | 1 |
| **9** | 51 | Male | Caucasian | HNSCC | Not recorded |  | Not recorded | ß | 1 | 0 | 2 |
| **10** | 74 | Female | Caucasian | Ovarian serous/high grade carcinoma | Primary | NA | 1997 | 0 | 0 | 0 | 0 |
| **11** | 47 | Male | Caucasian | Rectal adenocarcinoma | Metastatic | Liver | 649 | 0 | 0 | 0 | 0 |
| **12** | 34 | Female | Caucasian | Desmoplastic small round cell sarcoma | Primary | NA | 341 | 0 | 0 | 0 | 0 |
| **13** | 77 | Female | Caucasian | Gastric adenocarcinoma | Primary | NA | 35 | 0 | 0 | 0 | 0 |
| **14** | 63 | Female | Caucasian | Ovarian granulosa cell tumor | Primary | NA | 78 | 0 | 0 | 0 | 0 |
| **15** | 68 | Female | Caucasian | Cholangio-  carcinoma | Primary | NA | 527 | 0 | 0 | 0 | 0 |
| **16** | 67 | Male | Caucasian | Pancreatic adenocarcinoma | Metastatic | Liver | 9 | 0 | 2 | 0 | 4 |
| **17** | 56 | Female | Caucasian | Ovarian serous/high grade carcinoma | Primary | NA | 1597 | 0 | 1 | 0 | 2 |
| **18** | 66 | Female | Caucasian | Gastric adenocarcinoma | Metastatic | Colon | 1910 | 0 | 0 | 0 | 0 |
| **19** | 57 | Female | Caucasian | Colorectal cancer | Metastatic | Soft tissue | 554 | 0 | 0 | 0 | 0 |
| **20** | 48 | Male | Caucasian | Colon adenocarcinoma | Primary | NA | 201 | 0 | 0 | 0 | 0 |
| **21** | 49 | Female | Caucasian | Vulvar carcinoma | Metastatic | Liver | 13 | 0 | 0 | 0 | 0 |
| **22** | 57 | Male | Caucasian | Pancreatic ductal adenocarcinoma | Primary | NA | 710 | 0 | 0 | 0 | 0 |
| **23** | 59 | Female | Caucasian | Breast cancer (luminal B) | Primary | NA | 3591 | 0 | 0 | 0 | 0 |
| **24** | 48 | Female | Caucasian | Breast cancer (luminal B) | Metastatic | Liver | 505 | 0 | 0 | 0 | 0 |
| **25** | 57 | Male | Caucasian | Squamous NSCLC | Metastatic | Lymph Node | 4632 | 0 | 0 | 0 | 0 |
| **26** | 58 | Female | Caucasian | Breast cancer (ductal) | Metastatic | Liver | 37 | 0 | 0 | 0 | 0 |
| **27** | 66 | Male | Caucasian | Pancreatic adenocarcinoma | Primary | NA | 884 | 0 | 0 | 0 | 0 |
| **28** | 50 | Female | Caucasian | Cervical cancer (squamous) | Primary | NA | 123 | 0 | 0 | 0 | 0 |
| **29** | 42 | Male | Caucasian | Chordoma | Primary | NA | 323 | 0 | 0 | 0 | 0 |
| **30** | 50 | Female | Caucasian | Breast cancer (luminal B) | Metastatic | Liver | 333 | 0 | 0 | 0 | 0 |
| **31** | 26 | Female | Caucasian | Osteoblastic osteosarcoma | Metastatic | Lung | 12 | 0 | 0 | 0 | 0 |
| **32** | 59 | Female | Caucasian | Colon adenocarcinoma | Primary | NA | 242 | 0 | 0 | 0 | 0 |
| **33** | 49 | Female | Caucasian | Breast cancer (luminal A) | Metastatic | Liver | 2033 | 0 | 0 | 0 | 0 |
| **34** | 60 | Male | Caucasian | HNSCC (nasopharynx) | Metastatic | Lymph Node | 250 | 0 | 0 | 0 | 0 |
| **35** | 54 | Female | Caucasian | Breast cancer (ductal) | Metastatic | Liver | 698 | 0 | 0 | 0 | 0 |
| **36** | 66 | Male | Caucasian | Colon adenocarcinoma | Primary | NA | 61 | 0 | 0 | 0 | 0 |
| **37** | 40 | Female | Caucasian | Mesothelioma | Primary | NA | 12 | 0 | 0 | 0 | 0 |
| **38** | 52 | Male | Caucasian | Colon adenocarcinoma | Primary | NA | 12 | 0 | 0 | 0 | 0 |
| **39** | 52 | Female | Caucasian | Pancreatic neuroendocrine tumor | Metastatic | Liver | 527 | 0 | 0 | 0 | 0 |
| **40** | 63 | Male | Caucasian | Pancreatic adenocarcinoma | Primary | NA | 336 | 15 | 5 | 2 | 31 |
| **41** | 59 | Male | Caucasian | Small cell sarcoma | Primary | NA | 66 | 0 | 0 | 0 | 0 |
| **42** | 47 | Male | Caucasian | Nonseminomatous germ cell tumor | Metastatic | Lymph Node | 790 | 55 | 25 | 0 | 105 |
| **43** | 67 | Male | Caucasian | Gastric adenocarcinoma | Primary | NA | 210 | 10 | 65 | 25 | 215 |
| **44** | 47 | Female | Caucasian | Rectal adenocarcinoma | Primary | NA | 145 | 0 | 0 | 0 | 0 |
| **45** | 76 | Female | Caucasian | Chordoma | Primary | NA | 991 | 0 | 0 | 0 | 0 |
| **46** | 68 | Male | Caucasian | Uveal melanoma | Primary | NA | 1552 | 0 | 0 | 0 | 0 |
| **47** | 66 | Male | Caucasian | Colon adenocarcinoma | Primary | NA | Not recorded | 15 | 10 | 0 | 35 |
| **48** | 50 | Male | Caucasian | Colon adenocarcinoma | Primary | NA | 2408 | 0 | 0 | 0 | 0 |
| **49** | 41 | Male | Caucasian | Renal cell carcinoma | Primary | NA | 1061 | 0 | 0 | 0 | 0 |
| **50** | 64 | Female | Caucasian | Pancreatic adenocarcinoma | Primary | NA | Not recorded | 0 | 0 | 0 | 0 |
| **51** | 38 | Female | Caucasian | Breast cancer (ductal) | Primary | NA | 134 | 0 | 0 | 0 | 0 |
| **52** | 67 | Female | Caucasian | Breast cancer (triple negative) | Primary | NA | 105 | 0 | 0 | 0 | 0 |
| **53** | 60 | Male | Caucasian | Pancreatic adenocarcinoma | Primary | NA | 985 | 0 | 0 | 0 | 0 |
| **54** | 52 | Female | Caucasian | Colon adenocarcinoma | Metastatic | Liver | Not recorded | 0 | 0 | 0 | 0 |
| **55** | 58 | Female | Caucasian | Adenoid cystic carcinoma | Metastatic | Lung | 49 | 1 | 2 | 0 | 5 |
| **56** | 64 | Male | Caucasian | Colon adenocarcinoma | Primary | NA | 1555 | 0 | 5 | 0 | 10 |
| **57** | 75 | Male | Caucasian | Colon adenocarcinoma | Primary | NA | 1888 | 0 | 0 | 0 | 0 |
| **58** | 52 | Male | Caucasian | Pancreatic adenocarcinoma | Metastatic | Liver | 24 | 10 | 75 | 0 | 160 |
| **59** | 71 | Male | Caucasian | Colon adenocarcinoma | Primary | NA | 1413 | 0 | 0 | 0 | 0 |
| **60** | 56 | Female | Caucasian | Porocarcinoma | Metastatic | Lymph Node | 119 | 40 | 20 | 0 | 80 |
| **61** | 52 | Female | Caucasian | Breast cancer (ductal) | Primary | NA | 21 | 0 | 0 | 0 | 0 |
| **62** | 61 | Male | Caucasian | Bladder urothelial carcinoma | Primary | NA | Not recorded | 0 | 0 | 0 | 0 |
| **63** | 55 | Female | Caucasian | Ovarian serous/high grade carcinoma | Primary | NA | 1085 | 0 | 0 | 0 | 0 |
| **64** | 57 | Male | Caucasian | Bladder urothelial carcinoma | Primary | NA | 1178 | 0 | 0 | 0 | 0 |
| **65** | 70 | Female | Caucasian | Vulvar squamous cell carcinoma | Metastatic | Lymph Node | 484 | 8 | 12 | 3 | 41 |
| **66** | 58 | Female | Caucasian | Pancreatic cancer (squamous) | Metastatic | Liver | 659 | 0 | 0 | 0 | 0 |
| **67** | 40 | Male | Caucasian | Sarcoma | Metastatic | Other | 7 | 0 | 0 | 0 | 0 |
| **68** | 59 | Female | Caucasian | Rectal cancer | Primary | NA | 916 | 0 | 0 | 0 | 0 |
| **69** | 48 | Female | Caucasian | Ovarian adenocarcinoma | Metastatic | Ovary | Not recorded | 0 | 0 | 0 | 0 |
| **70** | 80 | Male | Caucasian | Bladder urothelial carcinoma | Metastatic | Bone | 56 | 2 | 1 | 0 | 4 |
| **71** | 78 | Female | Caucasian | Ovarian serous/high grade carcinoma | Metastatic | Other | Not recorded | 0 | 0 | 0 | 0 |
| **72** | 77 | Male | Caucasian | Cholangio-  carcinoma | Primary | NA | 169 | 0 | 0 | 0 | 0 |
| **73** | 84 | Male | Caucasian | Squamous NSCLC | Primary | NA | 679 | 0 | 0 | 0 | 0 |
| **74** | 57 | Female | Caucasian | Rectal adenocarcinoma | Primary | NA | 295 | 0 | 0 | 0 | 0 |
| **75** | 63 | Female | Caucasian | Pancreatic adenocarcinoma | Metastatic | Liver | 24 | 0 | 0 | 0 | 0 |
| **76** | 68 | Female | Caucasian | Sarcoma | Metastatic | Soft tissue | 284 | 1 | 1 | 0 | 3 |
| **77** | 72 | Male | Caucasian | Colon cancer | Primary | NA | 648 | 0 | 0 | 0 | 0 |
| **78** | 37 | Male | Caucasian | Esthesio-  neuroblastoma | Primary | NA | 469 | 0 | 0 | 0 | 0 |
| **79** | 65 | Male | Caucasian | Colorectal cancer | Primary | NA | 63 | 0 | 0 | 0 | 0 |
| **80*** | 50 | Female | Caucasian | Rectal cancer | Primary | NA | 258 | 0 | 0 | 0 | 0 |
| **80*** | 50 | Female | Caucasian | Rectal cancer | Metastatic | Ovary | 125 | 0 | 0 | 0 | 0 |
| **81** | 55 | Female | Caucasian | Breast cancer | Metastatic | Lymph Node | 2225 | 5 | 15 | 35 | 140 |
| **82** | 61 | Female | Caucasian | Pancreatic cancer | Metastatic | Ovary | 73 | 0 | 0 | 0 | 0 |
| **83** | 66 | Female | Caucasian | Pancreatic cancer | Metastatic | Liver | 49 | 0 | 0 | 0 | 0 |
| **84** | 71 | Female | Caucasian | Pancreatic cancer | Metastatic | Liver | 33 | 0 | 0 | 0 | 0 |
| **85** | 58 | Male | Caucasian | Gastric cancer | Primary | NA | 515 | 0 | 0 | 0 | 0 |
| **86** | 30 | Male | Caucasian | Uveal melanoma | Primary | NA | 313 | 0 | 10 | 0 | 20 |
| **87** | 77 | Male | Caucasian | Gastric cancer | Primary | NA | 554 | 35 | 32 | 3 | 108 |
| **88** | 54 | Female | Caucasian | Colon cancer | Primary | NA | 469 | 0 | 0 | 0 | 0 |
| **89** | 78 | Male | Caucasian | Bladder urothelial carcinoma | Metastatic | Kidney | 290 | 0 | 0 | 0 | 0 |
| **90** | 39 | Male | Caucasian | HNSCC (nasopharynx) | Metastatic | Liver | 75 | 0 | 0 | 0 | 0 |
| **91** | 77 | Male | Caucasian | Pancreatic cancer | Primary | NA | 1179 | 0 | 0 | 0 | 0 |
| **92** | 63 | Male | Caucasian | Adenoid cystic carcinoma  (salivary glands) | Metastatic | Soft tissue | 1354 | 10 | 15 | 10 | 70 |
| **93** | 51 | Female | Caucasian | Ovarian clear cell adenocarcinoma | Metastatic | Peritoneum/ abdominal wall | 920 | 2 | 0 | 0 | 2 |
| **94** | 52 | Male | Caucasian | Small-cell lung cancer | Primary | NA | 28 | 0 | 1 | 0 | 2 |
| **95** | 65 | Male | Caucasian | Melanoma | Metastatic | Lymph Node | 587 | 0 | 0 | 0 | 0 |
| **96** | 54 | Female | Caucasian | Colon cancer | Metastatic | Kidney | 149 | 0 | 0 | 0 | 0 |
| **97** | 58 | Female | Caucasian | Endometrial cancer | Metastatic | Mediastinum | Not recorded | 0 | 0 | 0 | 0 |
| **98** | 56 | Female | Caucasian | Ovarian carcinoma | Primary | NA | 266 | 35 | 10 | 0 | 55 |
| **99** | 51 | Female | Caucasian | Ovarian carcinoma | Metastatic | Skin | 741 | 0 | 0 | 0 | 0 |
| **100** | 44 | Female | Caucasian | Ovarian carcinoma | Metastatic | Other | 160 | 0 | 0 | 0 | 0 |
| **101** | 44 | Female | Caucasian | Squamous NSCLC | Primary | NA | 167 | 0 | 25 | 75 | 275 |
| **102** | 68 | Female | Caucasian | Non-NSCLC lung cancer | Primary | NA | 859 | 0 | 0 | 0 | 0 |
| **103** | 58 | Male | Caucasian | Squamous NSCLC | Primary | NA | 268 | 0 | 0 | 0 | 0 |
| **104** | 57 | Female | Caucasian | Synovial sarcoma | Primary | NA | 337 | 0 | 0 | 0 | 0 |
| **105** | 42 | Male | Caucasian | HNSCC | Primary | NA | 131 | 15 | 25 | 0 | 65 |
| **106** | 64 | Male | Caucasian | Squamous NSCLC | Metastatic | Liver | 70 | 0 | 0 | 0 | 0 |
| **107** | 75 | Female | Caucasian | Ovarian serous/high grade carcinoma | Primary | NA | 824 | 0 | 0 | 0 | 0 |
| **108** | 56 | Female | Asian | Esophageal cancer | Metastatic | Lung | 44 | 7 | 3 | 2 | 19 |
| **109** | 57 | Male | Caucasian | Esophageal cancer | Primary | NA | 1145 | 0 | 0 | 0 | 0 |
| **110** | 58 | Male | Not reported | Melanoma | Metastatic | Lymph Node | 297 | 0 | 0 | 0 | 0 |
| **111** | 70 | Male | Caucasian | Bladder urothelial carcinoma | Not recorded | Not recorded | 606 | 0 | 0 | 0 | 0 |
| **112** | 63 | Male | Caucasian | Duodenal cancer | Primary | NA | 1114 | 0 | 0 | 0 | 0 |
| **113** | 41 | Male | Not reported | Renal cell carcinoma | Primary | NA | 752 | 0 | 0 | 0 | 0 |
| **114** | 64 | Female | Caucasian | Adenoid cystic carcinoma  (salivary glands) | Metastatic | Lung | 410 | 0 | 5 | 90 | 280 |
| **115** | 75 | Male | Caucasian | Melanoma | Metastatic | Chest wall | 35 | 0 | 0 | 0 | 0 |
| **116** | 70 | Female | Caucasian | Ovarian serous/high grade carcinoma | Metastatic | Other | 2305 | 0 | 1 | 1 | 5 |
| **117** | 63 | Female | Caucasian | Ovarian carcinoma | Metastatic | Peritoneum/ abdominal wall | 215 | 0 | 5 | 95 | 295 |
| **118** | 71 | Male | Caucasian | Sarcoma (MPNST) | Metastatic | Lung | 121 | 0 | 0 | 0 | 0 |
| **119** | 74 | Male | Caucasian | Mesothelioma | Metastatic | Lung | 1293 | 0 | 0 | 0 | 0 |
| **120** | 74 | Male | Caucasian | Bladder urothelial carcinoma | Metastatic | Prostate | 1267 | 0 | 0 | 0 | 0 |
| **121** | 59 | Male | Caucasian | Renal cell carcinoma | Primary | NA | 825 | 0 | 0 | 0 | 0 |
| **122** | 43 | Female | Caucasian | Renal cell carcinoma (non-clear cell) | Primary | NA | 238 | 0 | 0 | 0 | 0 |
| **123** | 46 | Female | Caucasian | Ovarian serous/high grade carcinoma | Metastatic | Lymph Node | 373 | 0 | 0 | 0 | 0 |
| **124** | 59 | Male | Caucasian | Renal cell carcinoma | Metastatic | Lung | 909 | 0 | 0 | 0 | 0 |
| **125** | 55 | Female | Caucasian | Ovarian serous/high grade carcinoma | Primary | NA | 759 | 5 | 25 | 5 | 70 |
| **126** | 45 | Female | Caucasian | Breast cancer | Metastatic | Peritoneum/ abdominal wall | 309 | 0 | 0 | 0 | 0 |
| **127** | 63 | Male | Caucasian | Pancreatic cancer | Metastatic | Liver | 146 | 0 | 2 | 0 | 4 |
| **128** | 37 | Male | Caucasian | Liposarcoma | Metastatic | Lung | 755 | 5 | 75 | 0 | 155 |
| **129** | 56 | Male | Caucasian | NSCLC adenocarcinoma | Primary | NA | 990 | 0 | 0 | 0 | 0 |
| **130** | 66 | Male | Asian | Esophageal adenocarcinoma | Primary | NA | 1073 | 0 | 0 | 0 | 0 |
| **131** | 51 | Female | Caucasian | Adenoid cystic carcinoma | Primary | NA | 1052 | 2 | 6 | 3 | 23 |
| **132** | 35 | Female | Caucasian | Ovarian carcinoma, other | Metastatic | Peritoneum/ abdominal wall | 99 | 0 | 0 | 0 | 0 |
| **133** | 58 | Male | Caucasian | Laryngeal neuroendocrine carcinoma | Metastatic | Lymph Node | 184 | 0 | 0 | 0 | 0 |
| **134** | 58 | Male | Caucasian | Esophageal cancer | Primary | NA | 990 | 1 | 2 | 0 | 5 |
| **135** | 59 | Male | Caucasian | NSCLC adenocarcinoma | Metastatic | Skin | 107 | 0 | 0 | 0 | 0 |
| **136** | 74 | Male | Caucasian | Prostate adenocarcinoma | Primary | NA | 4169 | 0 | 0 | 0 | 0 |
| **137** | 56 | Male | Caucasian | Prostate cancer (castrate-resistant) | Metastatic | Other | 31 | 0 | 0 | 0 | 0 |
| **138** | 67 | Female | Caucasian | Adenoid cystic carcinoma  (salivary gland) | Metastatic | Lung | 819 | 0 | 0 | 0 | 0 |
| **139** | 66 | Female | Caucasian | Pancreatic cancer | Metastatic | Lung | 95 | 40 | 0 | 0 | 40 |
| **140** | 35 | Female | Caucasian | Breast cancer | Primary | NA | 1072 | 0 | 0 | 0 | 0 |
| **141** | 68 | Male | Caucasian | Osteosarcoma | Metastatic | Soft tissue | 185 | 0 | 0 | 0 | 0 |
| **142** | 67 | Female | Caucasian | Ovarian serous/high grade carcinoma | Primary | NA | 2027 | 45 | 35 | 10 | 145 |
| **143** | 67 | Male | Caucasian | Esophageal cancer | Metastatic | Lymph Node | 2180 | 3 | 85 | 10 | 203 |
| **144** | 59 | Female | Caucasian | Leiomyosarcoma | Metastatic | Liver | 84 | 0 | 0 | 0 | 0 |
| **145** | 59 | Female | Caucasian | Ovarian serous/high grade carcinoma | Primary | NA | 1100 | 5 | 10 | 5 | 40 |
| **146** | 60 | Female | Caucasian | Ovarian serous/high grade carcinoma | Metastatic | Peritoneum/ abdominal wall | 228 | 2 | 4 | 0 | 10 |
| **147** | 63 | Female | Caucasian | Adenoid cystic carcinoma | Metastatic | Other | 3878 | 0 | 3 | 2 | 12 |
| **148** | 67 | Male | Caucasian | Pancreatic cancer | Primary | NA | 2077 | 0 | 0 | 0 | 0 |
| **149** | 73 | Male | Caucasian | Squamous NSCLC | Primary | NA | 287 | 40 | 25 | 5 | 105 |
| **150** | 56 | Female | Caucasian | Breast cancer | Metastatic | Liver | 19 | 0 | 0 | 0 | 0 |
| **151** | 71 | Male | Caucasian | Squamous NSCLC | Primary | NA | 613 | 15 | 30 | 30 | 165 |
| **152** | 78 | Female | Caucasian | Rectal cancer | Primary | NA | 974 | 4 | 5 | 1 | 17 |
| **153** | 37 | Male | Caucasian | Colon adenocarcinoma | Primary | NA | 184 | 0 | 0 | 0 | 0 |
| **154** | 70 | Male | Caucasian | Large cell neuroendocrine tumor | Metastatic | Liver | 957 | 0 | 0 | 0 | 0 |
| **155** | 50 | Female | Caucasian | Breast cancer | Metastatic | Liver | 566 | 0 | 0 | 0 | 0 |
| **156** | 70 | Female | Caucasian | Ovarian carcinoma | Metastatic | Lymph Node | 416 | 5 | 65 | 25 | 210 |
| **157** | 78 | Male | Caucasian | Squamous NSCLC | Primary | NA | 258 | 15 | 65 | 10 | 175 |
| **158** | 70 | Male | Caucasian | Adenoid cystic carcinoma  (salivary gland) | Primary | NA | 8 | 5 | 90 | 0 | 185 |
| **159** | 69 | Male | Caucasian | Bladder urothelial carcinoma | Primary | NA | 635 | 0 | 0 | 0 | 0 |
| **160** | 77 | Female | Caucasian | Endometrial cancer | Not recorded | Not recorded | Not recorded | 15 | 30 | 5 | 90 |
| **161** | 67 | Male | African or Black | Angiosarcoma | Metastatic | Other | Not recorded | 0 | 0 | 0 | 0 |
| **162** | 59 | Male | Caucasian | Esophageal adenocarcinoma | Not recorded | Not recorded | Not recorded | 0 | 0 | 0 | 0 |
| **163** | 70 | Female | Caucasian | Gastric cancer | Not recorded | Not recorded | Not recorded | 60 | 5 | 0 | 70 |
| **164** | 70 | Male | Caucasian | Esophageal cancer | Not recorded | Not recorded | Not recorded | 0 | 0 | 0 | 0 |
| **165** | 63 | Male | Caucasian | Esophageal cancer | Metastatic | Other | Not recorded | 0 | 0 | 0 | 0 |
| **166** | 41 | Female | Caucasian | Chondrosarcoma | Metastatic | Other | Not recorded | 0 | 0 | 0 | 0 |
| **167** | 75 | Male | Caucasian | Sarcoma  (solitary fibrous) | Not recorded | Not recorded | Not recorded | 0 | 0 | 0 | 0 |
| **168** | 29 | Male | Caucasian | Colorectal neuroendocrine carcinoma | Metastatic | Other | 15 | 0 | 0 | 0 | 0 |
| **169** | 28 | Male | Caucasian | Sarcoma (epitheloid) | Metastatic | Skin | Not recorded | 0 | 0 | 0 | 0 |
| **170** | 38 | Male | Caucasian | Esophageal cancer | Primary | NA | Not recorded | 5 | 10 | 50 | 175 |
| **171** | 62 | Female | Caucasian | Peritoneal cancer | Metastatic | Lymph Node | Not recorded | 1 | 2 | 92 | 281 |
| **172** | 70 | Male | Caucasian | Bladder urothelial carcinoma | Primary | NA | Not recorded | 0 | 8 | 0 | 16 |
| **173** | 55 | Female | Caucasian | Endometrial cancer | Not recorded | Not recorded | Not recorded | 0 | 0 | 0 | 0 |
| **174** | 60 | Male | Caucasian | Squamous cell carcinoma of the lacrimal drainage system | Metastatic | Head and neck | Not recorded | 0 | 0 | 0 | 0 |
| **175** | 61 | Female | Caucasian | Endometrial cancer | Primary | NA | Not recorded | 2 | 5 | 0 | 12 |
| **176** | 67 | Female | Caucasian | Non-NSCLC lung cancer | Primary | NA | Not recorded | 0 | 0 | 0 | 0 |
| **177** | 39 | Male | Caucasian | Synovial sarcoma | Primary | NA | Not recorded | 0 | 0 | 0 | 0 |
| **178** | 43 | Male | Caucasian | Sarcoma (epitheloid) | Primary | NA | Not recorded | 0 | 0 | 0 | 0 |
| **179** | 46 | Female | Caucasian | Leiomyosarcoma | Metastatic | Other | Not recorded | 0 | 0 | 0 | 0 |
| **180** | 37 | Male | Caucasian | Round cell liposarcoma | Metastatic | Other | Not recorded | 50 | 20 | 0 | 90 |
| **181** | 32 | Male | Caucasian | Adenoid cystic carcinoma | Primary | NA | Not recorded | 1 | 1 | 93 | 282 |
| **182** | 36 | Male | Asian | Adenoid cystic carcinoma | Primary | NA | Not recorded | 0 | 0 | 0 | 0 |
| **183** | 79 | Male | Caucasian | Squamous NSCLC | Primary | NA | Not recorded | 5 | 80 | 10 | 195 |
| **184** | 58 | Male | Caucasian | Esophageal cancer | Primary | NA | Not recorded | 0 | 0 | 0 | 0 |
| **185** | 65 | Male | Caucasian | Esophageal cancer | Metastatic | Esophagus | Not recorded | 0 | 0 | 100 | 300 |
| **186** | 55 | Female | Caucasian | Ovarian serous/high grade carcinoma | Primary | NA | Not recorded | 32 | 15 | 3 | 71 |
| **187** | 37 | Female | Caucasian | Bladder urothelial carcinoma | Metastatic | Lymph Node | Not recorded | 0 | 0 | 0 | 0 |
| **188** | 61 | Female | Caucasian | Synovial sarcoma | Metastatic | Soft tissue | 169 | 0 | 0 | 0 | 0 |
| **189** | 53 | Female | Not reported | Endometrial cancer | Primary | NA | 521 | 0 | 0 | 0 | 0 |
| **190** | 36 | Male | Caucasian | Esophageal cancer | Primary | NA | 101 | 3 | 70 | 2 | 149 |
| **191** | 49 | Male | Caucasian | Myxoid/round cell liposarcoma | Primary | NA | 2651 | 0 | 0 | 0 | 0 |
| **192** | 53 | Male | Caucasian | Myxoid liposarcoma | Metastatic | Brain | 27 | 0 | 0 | 0 | 0 |
| **193** | 60 | Female | Caucasian | Adenoid cystic carcinoma | Metastatic | Kidney | 555 | 0 | 2 | 0 | 4 |
| **194** | 74 | Male | Caucasian | Adenoid cystic carcinoma | Metastatic | Lung | 1645 | 3 | 90 | 5 | 198 |
| **195** | 44 | Female | Caucasian | Gastric cancer | Metastatic | Peritoneum/ abdominal wall | 65 | 0 | 0 | 0 | 0 |
| **196** | 51 | Female | Caucasian | Synovial sarcoma | Metastatic | Other | 17 | 0 | 0 | 0 | 0 |
| **197** | 34 | Female | Caucasian | Synovial sarcoma | Metastatic | Lung | 1244 | 0 | 98 | 0 | 196 |
| **198** | 50 | Male | Caucasian | Myxoid liposarcoma | Metastatic | Soft tissue | 175 | 10 | 65 | 0 | 140 |
| **199** | 39 | Female | Other | Liposarcoma | Primary | NA | 2381 | 9 | 1 | 0 | 11 |
| **200** | 31 | Female | Caucasian | Synovial sarcoma | Metastatic | Lung | 136 | 5 | 60 | 35 | 230 |

*Both samples are from the same patient (#80).

*H*-score, histo-score; HNSCC, head-and-neck squamous cell carcinoma; MAGE-A4, melanoma-associated antigen A4; NA, not applicable; NSCLC, non-small cell lung cancer

**Supplementary Fig. 1 ⎜MAGE-A4 expression by age of tumor sample at analysis: abundance (a) and *H*-score (b).**

**a b**
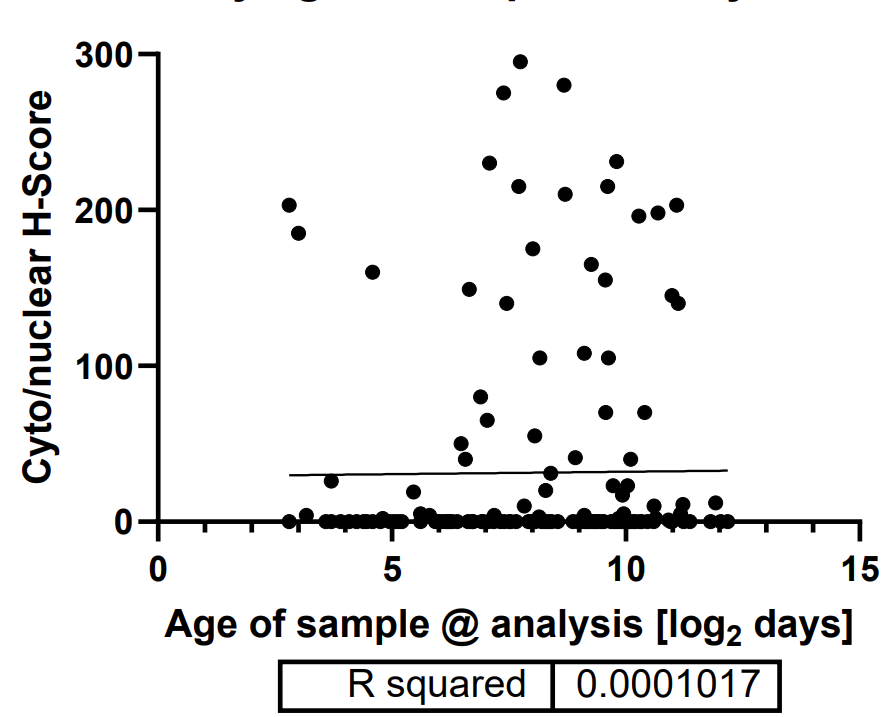

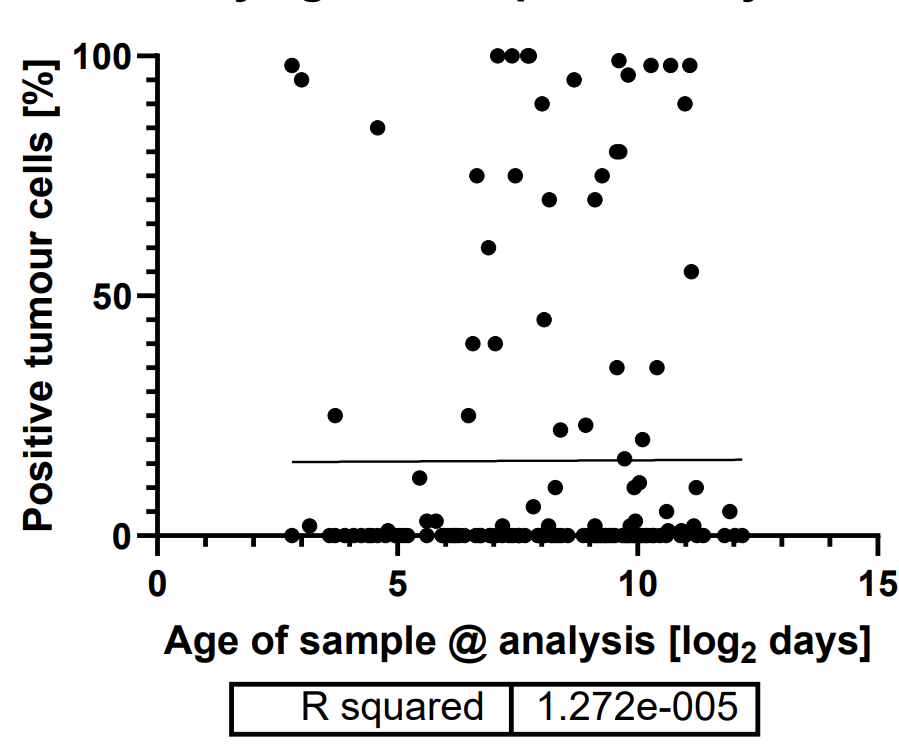


*H*-score, histo-score; MAGE-A4, melanoma-associated antigen A4.

**Supplementary Fig. 2 ⎜MAGE-A4 expression by tumor sample type (a) and gender (b).**

**a b**
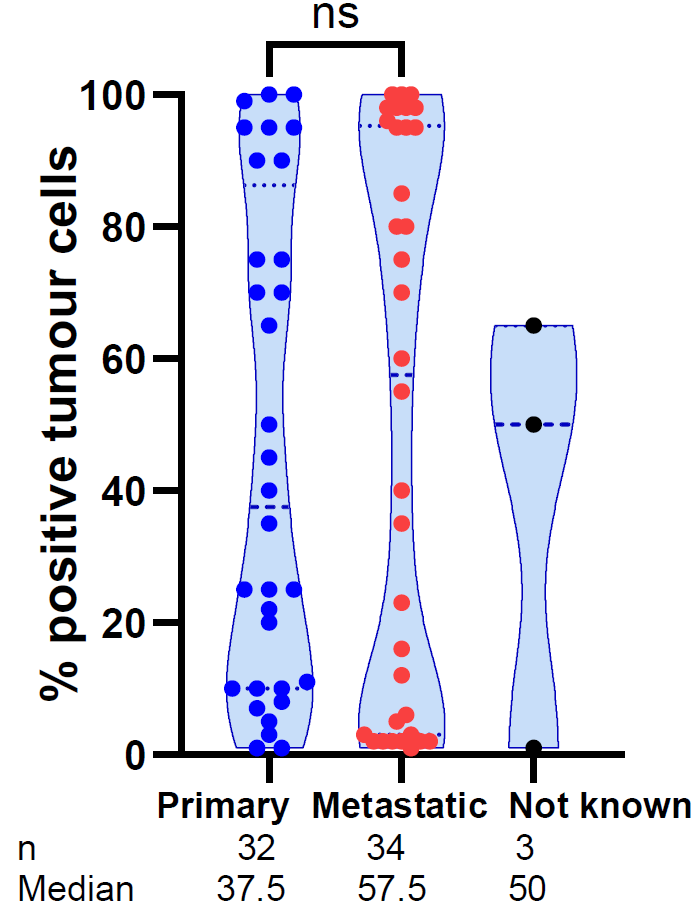

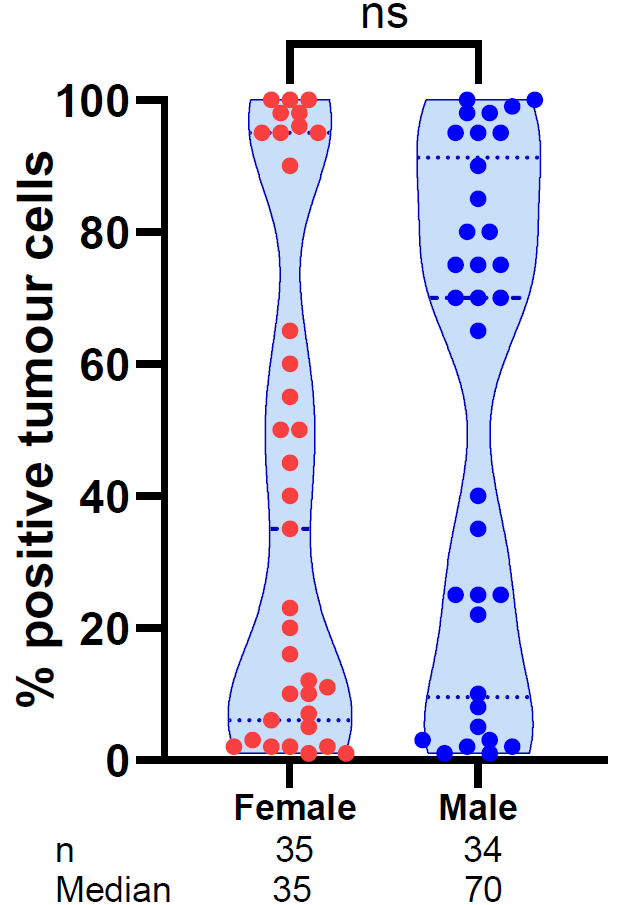


MAGE-A4, melanoma-associated antigen A4; ns, not significant.

**Supplementary Fig. 3 ⎜MHC class I and B2M protein expression in a subset of enrolled patients (n=19) who were MAGE-A4 positive and HLA-A*02:01 positive.**

In a total of 4 patients, 95% of tumor cells were positive for B2M and 90% of tumor cells were positive for MHC class 1. This is indicated by the red “4” in the larger data point.


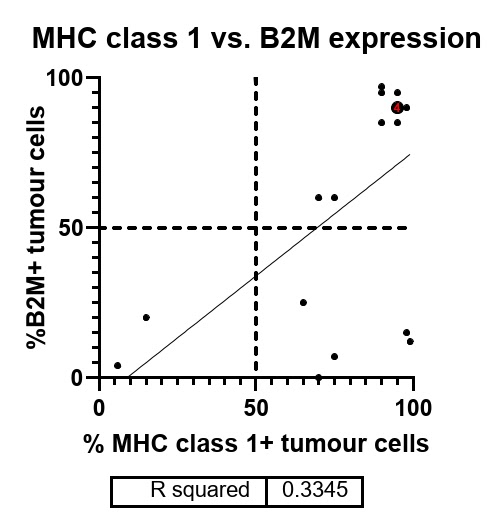


B2M, Beta-2-microglobulin; HLA-A*02:01, human leukocyte antigen-A*02:01; MHC, major histocompatibility; MAGE-A4, melanoma-associated antigen A4
